# Digital technologies in the COVID-19 responses in sub-Saharan Africa: policies, problems and promises

**DOI:** 10.11604/pamj.supp.2020.35.2.23456

**Published:** 2020-05-18

**Authors:** Elizabeth Bakibinga-Gaswaga, Stella Bakibinga, David Baxter Mutekanga Bakibinga, Pauline Bakibinga

**Affiliations:** 1 Legal Research Consortium, Kampala, Uganda; 2 African Population and Health Research Center, Nairobi, Kenya

**Keywords:** Digital technologies, COVID-19, sub-Saharan Africa, digital divide, 2030 agenda

## Abstract

The gains made five years after the adoption of the 2030 Agenda for Sustainable Development will be lost if the threats presented by the COVID-19 pandemic are not countered in a timely manner. The threat is worse in sub Saharan Africa where poverty and poor health and limited access to services present challenges to even the most robust of health systems on the continent. In light of the requisite public-private collaboration and multi-sectoral approach, digital technologies offer opportunities to support the COVID-19 responses. This commentary reviews the policy environment and the challenges presented by digital illiteracy, poor infrastructure, the high cost of installing ICT infrastructure, the volatile political environment and limited electricity supply as well as the opportunities that digital technologies provide to ensure that people and communities are still able to access goods and services. It highlights how digital technologies are being used by the governments, parliaments, judiciaries, schools, health service providers, transport authorities and marketers to reach their targeted audiences. The commentary concludes with recommendations on possible interventions that emphasize the need to address infrastructural limitations, promote public private partnerships and tackle the digital divide in all its dimensions, including from a gender and rural/urban perspective.

## Commentary

**Introduction: potential of digital technology in the Covid-19 response:** in September 2015, the global community committed to ‘leave no one behind’ and ‘reach the furthest behind first’ in the sustainable development agenda. These commitments have been matched with global, regional and national initiatives to transform lives, especially in the world’s least developed countries. The majority of people living on less than $1.90 a day live in sub-Saharan Africa (SSA) [1]. The gains made by countries since 2015 are under threat by the likely catastrophic impact of Covid-19, a global pandemic categorized as such by the World Health Organization (WHO). The numbers of confirmed cases in Africa have since soared past fifty thousand and they keep rising each passing day. This has prompted, countries and partner organizations to embark on various strategies to control the spread of the disease. These strategies have largely focused on sharing vital health information, including through the use of digital technologies to help implement stay-at-home directives. Because digital technologies including electronic tools, systems, devices and resources that generate, store or process data have the potential to transcend physical and, to a certain extent financial barriers, while providing real-time information, they have been embraced in various sectors [2]. Digital technologies create opportunities, increase democracy, popular participation and support socio-economic growth, among other benefits. Conversely, inadequacy of digital technologies reduces efficiency throughout the economy, diminishes the effectiveness of investment in priority sectors and development programs, causes a comparative disadvantage in trade and investment and lowers the quality of life [3]. In the health sector, digital health innovations have proven to be useful in clinical management of patients, record keeping, data collection and analysis, disease surveillance and support to health workers [4]. This commentary reflects on current policies, problems involved in reaching everyone and the potential digital technologies offer to ensure that no one is left behind during the responses to COVID-19 pandemic.

**Policy environment:** the digital transformation of societies is dependent upon the level of e-readiness, ‘how well a society is positioned to utilize the opportunities provided by Information and Communication Technologies (ICT)’ [2]. Internet penetration, the capacity of human capital, ICT infrastructure, policies and regulations, are equally critical measures of e-readiness. Countries that are not e-ready are not able to benefit from the opportunities presented by ICT. A 2017 report commissioned by the United Nations Conference on Trade and Development (UNCTAD) warned that digitalization was likely to widen income inequalities and that in a number of African countries, (including the Central African Republic, Eritrea and South Sudan), mobile cellular services still reach less than a third of the population [5]. The report observed that preparing for the digital economy requires a concerted, holistic, cross-sectoral and multi-stakeholder approach to national policy making and that most African countries also lack statistics on key aspects of the digital economy, hampering the ability to formulate evidence-based policies in this area [5].

**Problems:** critical challenges affecting the successful roll-out and use of digital innovations in LMICs, and SSA in particular, are a reflection of the political, social and cultural contexts in which these solutions are implemented. In the response to Covid-19, the digital divide made even more obvious by the failure of information on some of the proposed measures to reach intended audiences, is increasing socioeconomic disparities and health inequities worldwide, and more specifically in Africa. The World Literacy Foundation as reported that Africa leads the world in the percentage of the population without Internet connection at 88% and that in the countries that are connected, male Internet users outnumber their female counterparts in every region of the world. The gender digital divide and illiteracy hampers governments’ efforts to communicate critical public health to these pockets of the population and to counter misinformation. Most countries in Africa are not e-ready. Digital interventions that do not take into account limitation such as scarcity of steady power supply, lack of basic ICT skills by users, low network coverage, stringent government laws about access and use of social media, among others [6] are likely to fail. Additionally, failure to maintain the delicate balance between public health management and privacy interests exposes countries, especially those that have not enacted and do not enforce privacy and data protection legislation to litigation.

*Poor policy environments:* a number of countries in the WHO Afro region have developed strategies that have been followed by ICT and e-health policies. However, the adoption of policies in countries varies and where they exist, they are not operationalized. This has resulted in the mushrooming of privately and/or donor funded digital innovations that have not been scaled up beyond pilot stages. In some countries, strict government regulations on access and use of digital platforms have resulted in losses of previously reported gains. For example in Uganda, following the introduction of a social media tax, the use of mobile money and social media platforms reduced significantly with losses in tax revenue reported by the national tax regulatory body. In other cases, inadequacy of policy arises from conflict between regulatory and professional bodies. Although many countries in the sub region have e-health policies, there is a significant disconnect between policies and professional bodies resulting in challenges with implementation.

*Digital illiteracy:* digital literacy refers to an individual’s ability to find, evaluate, and compose clear information through writing and other media on various digital platforms. Digital literacy is evaluated by an individual’s grammar, composition, typing skills and ability to produce text, images, audio and designs using technology. Functional illiteracy in SSA exacerbates digital illiteracy because users’ inability to read and write to a level necessary to manage daily living and employment tasks that require reading skills beyond a basic level hampers effective communication of government’s measures to address COVID-19 and to counter misinformation campaigns.

*Poor ICT infrastructure, network coverage and limited access to digital devices:* these challenges need to be resolved for digital interventions to be effectively used to improve overall health and wellbeing. Otherwise, if the status quo is maintained, socioeconomic disparities and health inequities will continue to grow, leaving many deserving beneficiaries behind.

*Lack of energy:* ICT infrastructure and the equipment by the end users rely on the stable and sustainable supply of energy. More than half of Sub-Saharan Africa population lack access to electricity which renders most ICT equipment acquired redundant and hampers the dissemination of public health information and the delivery of online education to the intended recipients.

*The high cost of installing ICT infrastructure:* nearly all SSA states liberalized the telecommunication sector which allows private companies and foreign investors to provide ICT services at times at exorbitant rates. The governments have not subsidized these services to enable the ordinary citizens access them which is counter-productive in a crisis like the Covid-19 pandemic that is dependent upon dissemination to limit the risk to the public is counter-productive.

*The volatile political environment:* the political environment in most SSA is highly volatile and the democracy fragile. The majority of the population are youth who are unemployed but desire a better life hence unsettled. When they agitate for change they rely of social media platforms supported by the limited ICT infrastructure. The governments have in turn responded by shutting down the ICT services causing ICT ‘blackouts’ aimed at securing the state but at a high cost for those who rely on those platforms for medical services, livelihood and education.

**The promises: ICT-driven mechanisms to counter Covid-19:** countries faced with the rapid pace at which Covid-19 spread worldwide and informed by the many benefits of ICT have resorted to the available ICT means to address issues and mitigate risks as required. The nature of the pandemic dictates that interventions have to and will cover socio-economic and political spheres of life. In the socio-economic spheres, ICT interventions are required to provide solutions to health, education, trade, industry, communication, banking and labor subsectors. Politically, ICT interventions have been required for mobilization, policy making, governance and enforcement.

*Interventions for health:* the majority of digitally-enabled responses to Covid-19 have been in the health sector, with emphasis on awareness raising and training. Ministries of health have partnered with telecommunications companies to share mass education messages. Through these partnerships, surveillance is done as contacts are digitally monitored for their geolocations and symptoms. Hotlines have been established for reporting suspected cases or to seek clarifications. People have also been encouraged to call and consult with their healthcare providers before they go to health centres. This is to reduce overcrowding and keep health facilities open for those who absolutely need them as many have suspended elective and preventive procedures. In Ghana drones are being used to deliver the COVID-19 test kits and receive samples to and from hard to reach rural areas. WHO is using its online platform to further support the capacity of health workers in Africa through online courses [7] and tabletop or simulation exercises. WHO is helping local authorities craft radio messaging and TV spots to inform the public about the risks of COVID-19 and what measures should be taken, helping to counter disinformation and guiding countries on setting up call centres to ensure the public is informed [8].

*Interventions for socio-economic engagement:* in a bid to limit the spread of Covid-19, governments worldwide but more specifically in SSA have encouraged the public to use digital banking and purchasing platforms. Working with governments, banks and mobile telecommunication companies have waived and/or reduced fees for online and mobile banking services while increasing credit limits. Increasingly, more online shopping services have been made available reducing the need for people to go to crowded stores. Utility service providers for services such as water and electricity have opted for only online or mobile money payments. Meetings and other forms of social interaction have shifted to virtual platforms in the form of teleconferences. Policies for telecommuting have been operationalized to reduce the numbers of people commuting to workplaces.

*Interventions for education:* with an increasing number of states, provinces and even whole countries having closed institutions of learning as a response to the COVID-19 pandemic, over 80% of the world’s students are not attending school [9]. There is uncertainty about how long the lockdown of educational institutions will last thus the need to rely on the online learning platforms that have been availed by public and private sector stakeholders such as UNESCO [9].

*Interventions for access to justice:* justice has to be accessed in a timely manner and this remains critical even during the Covid-19 lockdown. Heads of the justice sector across Africa have permitted adaptation of operating procedures to ensure that judicial services can still be delivered. To this effect, court process is transmitted through email and freeware, cross-platform messaging services such as WhatsApp and judgments or rulings to be delivered via videolink or online forums.

*Interventions for governance:* legislative business cannot continue as usual in the face of the pandemic. Legislative bodies have continued to meet, albeit with proper social distancing measures in place including arrangements for legislators to sit in shifts. In contrast, the European Parliament has temporarily allowed electronic voting by email during the COVID-19 pandemic.

*Interventions for goods and services:* a number of companies in the goods and services industries have invested in applications (Apps) and created platforms that enable their customers to access their services. This is common with large supermarkets, food chains or food stores. The customer logs on, places their goods in a virtual cart and pays electronically and the goods are then delivered by the company courier to the customer’s door step. This is in line with social distancing and staying at home approaches in the fight against coronavirus. Although these innovations are working for some sections of the population, many people in low and middle income countries, including sub Saharan Africa are being left behind. These include, remote rural populations and the urban poor who are considered to have access to services due to their proximity to service providers in comparison to rural communities, yet this is not always the case.

**Promises: leaving no one behind-necessary actions:** as the WHO reaffirms, weak health systems cannot expect to use digital health appropriately, in the path to universal health coverage [4]. This calls for system-wide interventions to allow for the requisite flow of information and to build legal, policy and institutional frameworks and infrastructure required to ensure that health systems can respond to pandemics.

*Tackling the digital divide:* overall, it is important to tackle the digital divide. UNESCO has proposed that for education, this would require looking at issues related to access, teacher preparedness, and school-family communication. Before and after school closures, public-private partnerships could help ensure that all students have access to information technology, or to radio and television modalities [9] that are also relevant in some contexts and have been used successfully in crisis settings. Training teachers to use digital learning management systems and online learning pedagogy-before crises-is essential to transitioning to an online learning modality during a time of crisis [9]. Establishing communication lines between teachers and parents before crises and maintaining them as children learn from home is also key to support the most at risk children.

*Policy formulation:* better policies, informed by robust scientific evidence including e-readiness assessments for countries and regional blocs and tested for effectiveness, are a necessity if the region is to harness the benefits of ICT-aided interventions. Policies that create an enabling environment for stronger public-private partnerships and multi-stakeholder engagement will ensure that functions are streamlined.

*Sustainable electricity supply:* the success of digital interventions depends on access by all users to a reliable supply of electricity. A World Bank report, Electricity Access in Sub-Saharan Africa: uptake, reliability, and complementary factors for economic impact, identifies low uptake of available energy and inconsistency of supply as major challenges that will have to be addressed if digital solutions are to be used reliably [10].

*Investment into ICT infrastructure by governments:* while SSA governments have focused on the investment in roads and dams, it is high time ICT investments were prioritized and public interest taken into account so that the cost to the end user at the bottom of society is affordable. This will ensure that ‘no one is left behind’ as states seek to attain the SDG Agenda 2030.

## Conclusion

The mechanism(s) for the occurrence of mild or severe COVID-19 is related to an individual´s immune system, particularly the levels of pro-inflammatory responses. Genetic tolerance related to pregnancy may also have a role to play. Parasites, sex hormones, and constant exposure to pathogens may help lower the risk of pathological immune responses in SARS CoV-2 infections in immuno-competent individuals. Since the risk of severe disease is high among the elderly and those with chronic diseases, community testing for the virus must prioritise these groups of people in the light of shortage of testing kits. This will ensure that those found positive receive appropriate medical attention before they show any symptoms.

## Competing interests

The author declares no competing interests.

## References

[1] UNDP (2019). 2019 Human Development Index Ranking.

[2] Roztocki N, Piotr S, Weistroffer HR (2019). The role of information and communication technologies in socioeconomic development: towards a multi-dimensional framework. Information Technology for Development.

[3] Bakibinga EM (2004). Regulating Market Entry in the Telecommunications Sector in an Integrated East Africa: Towards a Common Licensing Framework in Norwegian Research Center for computers and law Faculty of Law 2004.

[4] World Health Organization (2019). WHO guideline: recommendations on digital interventions for health system strengthening.

[5] UNCTAD (2019). African Countries Need To Strengthen Their Digital Readiness To Benefit Fully From Digitalization.

[6] Betjeman TJ, Soghoian SE, Foran MP (2013). mHealth in Sub-Saharan Africa. International Journal of Telemedicine and Applications.

[7] WHO (2020). Amid lockdowns in Africa, WHO launches online training for COVID-19 responders.

[8] WHO (2020). Rolling updates on corona virus disease (COVID-19).

[9] UNESCO (2020). COVID-19 Educational Disruption and Response.

[10] Blimpo MP, Cosgrove-Davies M (2019). Electricity Access in Sub-Saharan Africa Uptake, Reliability, and Complementary Factors for Economic Impact, in Africa Development Forum series.

